# Supplementary material for: Shared genetic architecture between constipation and aging-related phenotypes: Insights from large-scale genome-wide cross-trait analysis
Source: Medicine (Baltimore). 2026 Oct 9;105(41):e51036. doi: 10.1097/MD.0000000000051036 (PMC13657532; doi:10.1097/MD.0000000000051036)
Supplement: Supplementary file 1 [file medi-105-e51036-s001.docx]

**Supplementary Methods S1. Core analytical models and parameter settings**

**Overview and common data processing**

This study was a cross-trait analysis of publicly available genome-wide association study (GWAS) summary statistics. Analyses were conducted for the FinnGen registry-derived constipation phenotype and four aging-related phenotypes: frailty index (FI), telomere length (TL), facial aging (FA), and GrimAge acceleration (GAA). Effect alleles were harmonized between paired datasets before cross-trait analyses. Variants with missing effect estimates, standard errors, allele-frequency information, or sample-size information were excluded where these variables were required. For colocalization analyses, FinnGen constipation summary statistics were lifted from GRCh38 to GRCh37, and paired datasets were analyzed using GRCh37 coordinates. European-ancestry linkage disequilibrium (LD) reference data were used throughout, as all source GWAS datasets were based predominantly on individuals of European ancestry.

Software versions are reported in the main Methods. The analytical settings and multiple-testing procedures for each method are summarized below.

**Linkage disequilibrium score regression**

Genome-wide genetic correlations between constipation and each aging-related phenotype were estimated using linkage disequilibrium score regression (LDSC, v1.0.1). Under LDSC, the expected product of z-scores for SNP j across two traits is modeled as:

*E(z₁ⱼz₂ⱼ) = c + [√(N₁N₂)/M] r₍g₎√(h₁²h₂²)lⱼ*

where z₁ⱼ and z₂ⱼ are association z-scores for the two traits, N₁ and N₂ are sample sizes, M is the number of SNPs, h₁² and h₂² are SNP-heritability estimates, lⱼ is the LD score of SNP j, r₍g₎ is the genetic correlation, and c is the cross-trait intercept. The intercept was estimated rather than constrained and was used to assess residual covariance potentially attributable to sample overlap or correlated confounding.

Analyses used HapMap3 SNPs and European LD scores. Genetic correlations were reported with standard errors and two-sided 95% confidence intervals calculated as r₍g₎ ± 1.96 × SE. Benjamini–Hochberg false discovery rate (BH-FDR) correction was applied across the four constipation–aging phenotype comparisons within LDSC.

**High-definition likelihood analysis**

High-definition likelihood (HDL, v1.4.0) was used as a complementary genetic-correlation analysis. HDL estimates genetic covariance using a likelihood framework that incorporates genome-wide LD information. Genetic correlation was defined as:

*r₍g₎ = σ₍g,12₎ / √(h₍g,1₎²h₍g,2₎²)*

where σ₍g,12₎ is the genetic covariance between traits 1 and 2, and h₍g,1₎² and h₍g,2₎² are their SNP heritabilities. HDL analyses used the UK Biobank imputed-SNP LD eigen-decomposition reference panel (Nref = 335,265). BH-FDR correction was applied separately across the four phenotype-pair comparisons within HDL. LDSC and HDL were interpreted as complementary estimates of genome-wide genetic correlation.

**PLACO analysis**

Pleiotropy analysis under the composite null hypothesis (PLACO) was used to identify locus-level cross-trait association signals. The composite null hypothesis was:

*H₀: β₁β₂ = 0*

where β₁ and β₂ denote the genetic effects of a variant on constipation and the corresponding aging-related phenotype, respectively. Thus, the null hypothesis includes variants associated with neither trait or with only one trait.

Variants with PPLACO < 5 × 10⁻⁸ were considered genome-wide significant in the primary analysis. Because four constipation–aging phenotype pairs were evaluated, PPLACO < 1.25 × 10⁻⁸ was additionally assessed as a cross-pair Bonferroni sensitivity threshold.

**FUMA locus definition and MAGMA gene-based analysis**

Genome-wide significant PLACO variants were annotated using FUMA (v1.5.2). Independent significant SNPs and lead SNPs were defined using LD thresholds of r² ≥ 0.6 and r² ≥ 0.1, respectively, based on the 1000 Genomes Phase 3 European reference panel. Genomic risk loci within 250 kb were merged. The extended major histocompatibility complex region was excluded. FUMA was used for locus definition and gene annotation and was not treated as an additional hypothesis-testing stage.

Gene-based analyses were performed using MAGMA v1.08 with the SNP-wise mean model, which aggregates SNP association statistics within a gene while accounting for LD between SNPs. Gene-level significance was assessed separately for each constipation–aging phenotype pair using Bonferroni correction:

*P₍Bonferroni₎ = 0.05 / N₍genes₎*

where N₍genes₎ denotes the number of genes with valid MAGMA results for that phenotype pair. The corresponding numbers of tested genes were 18,167 for constipation–FI, 18,504 for constipation–FA, 18,645 for constipation–TL, and 18,117 for constipation–GAA.

Gene-set analyses were conducted using MSigDB gene sets implemented through FUMA. BH-FDR and Bonferroni corrections were applied separately across the complete valid gene-set testing universe within each phenotype pair. MAGMA gene-property analyses tested whether gene-level association signals were associated with expression across 54 GTEx v8 tissues. Tissue-level BH-FDR correction was applied within each phenotype pair, and P < 9.26 × 10⁻⁴ (0.05/54) was displayed as an additional Bonferroni reference threshold.

**Bayesian colocalization analysis**

Bayesian colocalization was conducted using the coloc.abf model (coloc v5.2.1) within FUMA-defined genomic risk-locus boundaries. For each locus, variants shared by both GWAS datasets were retained after coordinate matching and allele harmonization.

The model compares five mutually exclusive hypotheses: H₀, no association with either trait; H₁, association with constipation only; H₂, association with the aging-related phenotype only; H₃, both traits are associated but with different causal variants; and H₄, both traits are associated with a shared causal variant.

Approximate Bayes factors were calculated using the default priors p₁ = 1 × 10⁻⁴, p₂ = 1 × 10⁻⁴, and p₁₂ = 1 × 10⁻⁵, where p₁ and p₂ are the prior probabilities that a variant is associated with each individual trait, and p₁₂ is the prior probability that a variant is associated with both traits. The coloc.abf model assumes at most one causal variant per trait within a tested region and does not explicitly incorporate an LD matrix into posterior-probability estimation. The 1000 Genomes Phase 3 European panel was used for regional LD annotation and visualization.

Posterior probability for H₄ was denoted as PP.H4. Loci with 0.5 < PP.H4 ≤ 0.7 were classified as exploratory signals, whereas loci with PP.H4 > 0.7 were considered to show stronger colocalization support within this study.

**Summary-data-based Mendelian randomization and HEIDI analysis**

Summary-data-based Mendelian randomization (SMR, v1.3.1) was performed using a whole-blood eQTL dataset and eQTLGen summary data. The SMR estimate can be expressed as a ratio estimate:

*β̂₍SMR₎ = β̂₍GWAS₎ / β̂₍eQTL₎*

where β̂₍GWAS₎ is the estimated association of the top cis-eQTL instrument with the phenotype, and β̂₍eQTL₎ is its estimated association with gene expression. SMR evaluates whether trait and expression associations are compatible with a shared variant-mediated association; it does not establish that gene expression causally mediates the trait association.

The HEIDI test was used to evaluate heterogeneity among instrumental variants. Retained SMR evidence required PHEIDI > 0.05. Within each phenotype-by-eQTL-resource analysis, PSMR values were corrected across all tested probes using BH-FDR. Corrected SMR evidence required an FDR-adjusted PSMR < 0.05 together with PHEIDI > 0.05. Bonferroni correction across all tested probes was additionally applied as a sensitivity analysis. Shared SMR evidence required the same gene/probe to meet the corrected SMR and HEIDI criteria for both constipation and the corresponding aging-related phenotype within the same eQTL resource.

**Multiple-testing strategy and interpretation**

BH-FDR correction was used for the four genetic-correlation tests within each method, all tested SMR probes within each phenotype-by-eQTL-resource analysis, MAGMA gene sets within each phenotype pair, and tissue tests within each phenotype pair. Bonferroni correction was used for MAGMA gene-based tests, as a sensitivity analysis for SMR, and as the cross-pair sensitivity threshold for PLACO. FUMA annotation was not regarded as an independent inferential stage. All tests were two-sided unless otherwise specified.
