# Supplementary material for: Shared genetic architecture between constipation and aging-related phenotypes: Insights from large-scale genome-wide cross-trait analysis
Source: Medicine (Baltimore). 2026 Oct 9;105(41):e51036. doi: 10.1097/MD.0000000000051036 (PMC13657532; doi:10.1097/MD.0000000000051036)
Supplement: Supplementary file 2 [file medi-105-e51036-s002.docx]

**Table 2: Genetic correlation between constipation and aging-related phenotypes**

| **Trait pairs** | **Method** | **r_g_(95%CI)** | ***p*** | ***p*_FDR** |
| --- | --- | --- | --- | --- |
| Constipation-FI | LDSC | 0.435(0.345 to 0.525) | 2.65x10^-21^ | **1.06x10^-20^*** |
|  | HDL | 0.476(0.377 to 0.576) | 5.57x10^-21^ | **2.23x10^-20^*** |
| Constipation-TL | LDSC | -0.087(-0.160 to -0.013) | 0.021 | **0.041*** |
|  | HDL | -0.101(-0.200 to -0.002) | 0.046 | 0.092 |
| Constipation-FA | LDSC | -0.007(-0.089 to 0.074) | 0.860 | 0.860 |
|  | HDL | -0.040(-0.118 to 0.038) | 0.314 | 0.419 |
| Constipation-GAA | LDSC | 0.043(-0.111 to 0.198) | 0.583 | 0.777 |
|  | HDL | -0.009(-0.181 to 0.162) | 0.916 | 0.916 |

Note: GAA, GrimAge acceleration; r_g_, genetic correlation; CI, confidence interval; FDR, false discovery rate. SEs are reported in Supplementary Tables S1–S2. Two-sided 95% CIs were calculated as r_g_ ± 1.96 × SE. BH-FDR correction was applied separately across the four phenotype pairs within each method, *BH-FDR < 0.05.
