# Supplementary material for: Shared genetic architecture between constipation and aging-related phenotypes: Insights from large-scale genome-wide cross-trait analysis
Source: Medicine (Baltimore). 2026 Oct 9;105(41):e51036. doi: 10.1097/MD.0000000000051036 (PMC13657532; doi:10.1097/MD.0000000000051036)
Supplement: Supplementary file 3 [file medi-105-e51036-s004.docx]

**Title Page**

**Shared genetic architecture between constipation and aging-related phenotypes: insights from large-scale genome-wide cross-trait analysis**

Yixuan Liao (MD)^1^, Wei Liu (MD)^3^, Xiaojin Liu (MD)^2^,Yingying Xiao (MD)^2^^,*^

^1^Department of Gastroenterology, Xiangya Hospital, Central South University, Changsha 410008, Hunan, China

^2^Department of Plastic and Cosmetic Surgery, Xiangya Hospital, Central South University, Changsha 410008, Hunan, China

^3^Department of Gastrointestinal Surgery, Xiangya Hospital, Central South University, Changsha 410008, Hunan, China

^*^Corresponding Author:

Yingying Xiao, E-mail: 13755014927@163.com

**List of abbreviations**

ATC = Anatomical Therapeutic Chemical

BH-FDR = Benjamini-Hochberg false discovery rate

CNS = central nervous system

eQTL = expression quantitative trait locus

FA = facial aging

FI = frailty index

FUMA = Functional Mapping and Annotation

GAA = GrimAge acceleration

GTEx = Genotype-Tissue Expression

GWAS = genome-wide association study

HDL = high-definition likelihood

HEIDI = heterogeneity in dependent instruments

ICD-10 = International Classification of Diseases, Tenth Revision

LD = linkage disequilibrium

LDSC = linkage disequilibrium score regression

LTL = leukocyte telomere length

MAGMA = Multi-marker Analysis of GenoMic Annotation

MHC = major histocompatibility complex

MSigDB = Molecular Signatures Database

PLACO = Pleiotropy Analysis under Composite Null Hypothesis

S-LDSC = stratified linkage disequilibrium score regression

SMR = summary-data-based Mendelian randomization

SNP = single-nucleotide polymorphism

TL = telomere length

**Shared genetic architecture between constipation and aging-related phenotypes: insights from large-scale genome-wide cross-trait analysis**

**Abstract**

**Background:** While the frequency of constipation increases sharply with age, the genetic links between constipation and aging-related phenotypes remain largely unexplored. So far, research has typically focused only on individual characteristics while neglecting broader genetic connections. In this study, we evaluated the shared genetic architecture of constipation and aging-related phenotypes in order to better understand the causes of aging-related intestinal dysfunction.

**Methods:** Using publicly available GWAS summary statistics, we developed a multi-stage analysis pipeline to identify common signals at the variation and genetic levels. We studied four aging-related phenotypes: frailty index (FI), telomere length (TL), facial aging (FA) and GrimAge acceleration (GAA). Our approach combines LDSC and HDL with PLACO, FUMA and Bayesian colocalization to identify pleiotropic loci, followed by tissue-specific mapping via MAGMA and S-LDSC.

**Results:** Constipation showed a moderate positive genetic correlation with FI in both LDSC and HDL. The inverse correlation with TL was weak and method-dependent. No significant genome-wide genetic correlation was detected with FA or GAA. Multi-layer locus mapping and gene-based analyses prioritized several candidate genes, including MSL2, STAG1, and SRSF9, through different combinations of association-based evidence. However, no gene showed multiple-testing-corrected SMR evidence shared between constipation and any of the four aging-related phenotypes. MAGMA suggested cerebellar enrichment for genes associated with the constipation–FI pair, whereas S-LDSC identified broader CNS enrichment for FI but no significant cell-type enrichment for constipation.

**Conclusions:** These findings support a shared genetic component between constipation and selected aging-related phenotypes and provide candidate loci, genes and tissues for further functional investigation.

**Keywords:**

Aging, frailty, constipation, GWAS, brain

**Introduction**

Constipation is a prevalent and bothersome gastrointestinal disorder, characterized by difficult defecation, hard and dry stools, and a reduced frequency of defecation^[1][2]^. Left unaddressed, constipation has been observationally associated with an increased risk of coronary heart disease, although residual confounding and reverse causation cannot be excluded^[3]^. It can also substantially impair physical, psychological, and social well-being, thereby reducing quality of life^[2]^. This multifaceted syndrome not only impairs physical health but also imposes considerable psychological and social burdens on patients^[3]^, further compromising their quality of life. Shifts in dietary patterns and lifestyle changes have fueled a rising global incidence of constipation in recent years^[4]^. According to the Rome IV criteria, its worldwide prevalence is approximately 10.1% - a figure that continues to climb^[5]^. This aging-related increase is significant because as people age, physiological changes tend to exacerbate gastrointestinal dysfunction. Notably, the prevalence of constipation rises sharply with age, reaching as high as 33.5% among people over 60 years old^[6][7]^. Insufficient dietary fiber intake - often linked to aging-related changes in eating habits - reduced physical activity, and age-induced alterations in neuromuscular structures are established risk factors for constipation in older adults^[8][9]^. This gap leaves us without a clear answer of why the elderly are disproportionately affected, hindering the development of targeted prevention and intervention strategies for this vulnerable group .

Genome-wide association study (GWAS) is an unbiased genetic approach^[10]^ that systematically identifies genomic variations (especially single nucleotide polymorphisms, SNPs) associated with diseases or complex traits^[11]^. It has demonstrated advantages in researching polygenic diseases such as diabetes and heart disease^[12]^. However, GWAS only reveals statistical associations rather than direct causal relationships, and the identified variants generally explain only part of trait heritability^[13][14]^. To address these challenges, a series of advanced computational methods have been developed in the field of genetics: Linkage Disequilibrium Score Regression (LDSC)^[15]^ and High-Definition Likelihood (HDL) can accurately estimate the genetic correlation between traits (with HDL achieving higher precision)^[16]^. To translate statistical associations into biological mechanisms, a multi-step post-GWAS framework is employed. Pleiotropy Analysis under Composite Null Hypothesis (PLACO)^[12]^ is used to identify shared genetic loci across multiple traits. Tools like Functional Mapping and Annotation (FUMA )are used to map associated loci to candidate genes^[17]^. Bayesian colocalization^[18]^ and Summary-data-based Mendelian randomization (SMR)^[19]^ are then used to assess whether association signals are compatible with a shared variant or gene-expression-associated signal. Furthermore, MAGMA provides a powerful suite of analyses, including gene-based tests (to aggregate SNP signals), geneset analysis (to identify pathways), and gene-property analysis (to test tissue-specific expression)^[20]^. These can be complemented by S-LDSC, which partitions overall trait heritability to identify etiologically relevant tissues^[21]^. We employed these integrated computational methods and large-scale GWAS data to explore the shared genetic architecture and potential pathways between constipation and aging, thereby providing new insights into the genetic underpinnings of the high incidence of constipation in older adults.

**Methods**

**Acquisition and curation of GWAS summary statistics**

Our analysis focused on constipation and four specific aging-related phenotypes representing different dimensions of aging: frailty index (FI), telomere length (TL), facial aging (FA) and GrimAge acceleration (GAA). For this analysis, we obtained GWAS summary statistics from publicly accessible datasets composed of individuals of European ancestry. GWAS summary statistics for constipation were obtained from FinnGen release 11^[22]^ using endpoint K11_CONSTIPATION. This registry-derived binary endpoint comprised 44,590 cases and 409,143 controls. According to the FinnGen endpoint definition, cases were identified from nationwide registers using ICD-10 code K59.0 in hospital-discharge or cause-of-death records and/or purchases of drugs classified under ATC code A06A, with a minimum of three qualifying registry events. Controls were FinnGen participants who did not meet this case definition^[23]^. Detailed characteristics of these five GWAS datasets are provided in Table1. The FI, TL, and FA GWASs shared UK Biobank participants, although the extent of overlap was unknown. No UK Biobank contribution was reported for constipation or GrimAge acceleration. To avoid false positive results and guarantee the reliability and accuracy of the GWAS datasets employed in this research, we ruled out all SNPs located within the major histocompatibility complex (MHC) on chromosome 6 (25Mb-35Mb), as this region is known to have a highly complex gene structure and strong linkage disequilibrium (LD).

**Genetic correlation assessment of constipation and aging-related phenotypes**

To evaluate the shared genetic architecture, we conducted a genome-wide genetic correlation analysis between constipation and the four specified age-associated phenotypes utilizing two complementary methods: LDSC and HDL analysis. LDSC was employed to estimate the overall genetic overlap between traits by regressing the association statistics of each SNP against its corresponding LD score. The HDL method was applied as a complementary approach to enhance statistical power and precision by more comprehensively utilizing genome-wide LD information. The genetic correlation coefficient, denoted as rg, quantifies the degree of shared genetic influence between two traits on a scale from -1 to 1. A positive rg value indicates that these traits share common genetic factors, while a negative rg value suggests an inverse genetic association between them. The study applied a False Discovery Rate (FDR) correction to address multiple testing. A significant genetic correlation between two traits was determined when the FDR-adjusted p-value (*p* FDR) was less than 0.05.

**Identification and validation of pleiotropic and genomic risk loci between constipation and aging-related phenotypes - PLACO, FUMA and Bayesian colocalization**

To identify specific shared genetic loci between constipation and aging-related traits, we began our analysis with PLACO-based pleiotropic analysis. PLACO was used to test for locus-level pleiotropic associations between constipation and each aging-related phenotype. Unlike LDSC and HDL, which estimate genome-wide genetic correlation, PLACO identifies specific loci showing joint association signals and therefore provides complementary locus-level evidence of shared genetic architecture. Variants with *P_PLACO_* < 5×10^-8^ were considered genome-wide significant. FUMA was subsequently applied to annotate the identified loci and map them to nearby or functionally supported genes using genomic annotations, eQTL information, and chromatin interaction data. Bayesian colocalization analysis was subsequently performed using the coloc.abf function for FUMA-defined genomic risk loci retained at the prespecified PLACO threshold. The analyzed interval corresponded to the FUMA start and end coordinates for each locus. FinnGen constipation summary statistics were lifted from GRCh38 to GRCh37, and all paired GWAS datasets were analyzed using GRCh37 coordinates. Within each region, variants present in both datasets and containing non-missing effect estimates, standard errors, allele frequencies, and sample-size information were retained after SNP and allele harmonization. Approximate Bayes factors were calculated using the default priors *p_1_*=1 × 10^-4^, *p_2_*=1 × 10^-4^, and *p_12_*=1 × 10^-5^. The coloc.abf model assumes at most one causal variant per trait within each region and does not explicitly incorporate an LD matrix into the posterior calculation; the European 1000 Genomes Phase 3 panel was used for regional LD annotation and visualization.

**SMR analysis of gene-expression and trait associations**

SMR integrates GWAS and eQTL summary statistics to evaluate whether gene-expression and trait associations are compatible with a shared variant-mediated association. Analyses were conducted using the whole-blood eQTL dataset and eQTLGen. The HEIDI test was used to assess heterogeneity in the instrumental-variable associations, with *P_HEIDI_* > 0.05 required for retained SMR evidence. Within each phenotype-by-eQTL dataset analysis, *P_SMR_* values were corrected across all tested probes using BH-FDR, with Bonferroni correction applied as a sensitivity analysis. Corrected SMR evidence required an FDR-adjusted *P_SMR_* < 0.05 together with *P_HEIDI_* > 0.05. Evidence was considered shared only when the same probe met the corrected SMR and HEIDI criteria for both constipation and the corresponding aging-related phenotype.

**Gene-based analysis and functional annotation using MAGMA**

To transition from individual SNP associations to a gene-centric perspective, we utilized MAGMA to aggregate the signals from all SNPs within a gene. This gene-based analysis aggregated SNP-level associations into gene-level statistics. After restricting the MAGMA output to autosomal genes with finite positive *p* values, the numbers of genes with valid results were 18,167 for constipation–FI, 18,504 for constipation–FA, 18,645 for constipation–TL, and 18,117 for constipation–GAA. Bonferroni correction was applied separately within each phenotype pair using 0.05 divided by the corresponding number of valid gene tests. MAGMA accounted for linkage disequilibrium between variants, and gene-level significance was evaluated using the pair-specific Bonferroni thresholds. After identifying shared genes, we conducted a MAGMA gene-set analysis to uncover the biological processes in which these genes are collectively involved. MAGMA gene-set analysis was performed using the gene sets available in the FUMA implementation of MSigDB. Depending on the trait pair, 17,000-17,002 gene sets with valid results were tested. Benjamini-Hochberg FDR and Bonferroni corrections were applied separately across the complete gene-set testing universe within each constipation–aging phenotype pair. Gene sets with (*P_FDR_* < 0.05) were considered FDR significant. Finally, to understand the physiological contexts in which these genes operate, we performed a tissue-specific enrichment analysis to pinpoint the biological context of the trait. This analysis utilized gene expression data across 54 human tissues from the GTEx project to determine where trait-associated genes are predominantly active.

**Validating tissue-specific signals with S-LDSC**

As a complementary and distinct approach to MAGMA, we employed S-LDSC to identify tissues of etiological relevance. Unlike MAGMA, which tests if associated genes are expressed in a tissue, S-LDSC partitions the total genome-wide heritability of each trait, testing whether heritability is significantly enriched in genomic regions corresponding to genes preferentially expressed in specific tissues. A key advantage is its ability to control for confounding from polygenic effects, population stratification, and polygenicity, enabling robust inference of tissue-specific genetic contributions to the trait’s architecture using only summary-level data, without the need for individual-level genotype data.

**Multiple-testing correction and evidence thresholds**

For LDSC and HDL, BH-FDR correction was applied separately across the four constipation–aging phenotype correlations within each method. PLACO variants meeting *P_PLACO_* < 5 × 10^-8^ were considered genome-wide significant. Because four phenotype pairs were examined, *P_PLACO_* < 1.25 × 10^-8^ was additionally evaluated as a cross-pair Bonferroni sensitivity threshold. FUMA was used for locus definition and functional annotation and was not treated as a separate hypothesis-testing stage. Colocalization results were classified as low-confidence exploratory evidence for 0.5 < *PP.H4* ≤ 0.7 and stronger evidence within this study for *PP.H4* > 0.7. Tissue enrichment analyses were corrected across tested tissues using BH-FDR. For the MAGMA gene-property analysis, a Bonferroni -corrected threshold of *p* < 9.26 × 10^-4^ (0.05/54 GTEx tissues) was additionally displayed as a more stringent reference.

**Software and packages**

All primary statistical analyses were performed using R software (version 4.2.2). LDSC and S-LDSC analysis made use of "LDSC" software (v1.0.1)^[15]^. For PLACO analytical approach between constipation and age-associated phenotypes, the "PLACO" package was utilized^[12]^. In this study, Bayesian colocalization analysis relied on the "coloc" package (version 5.2.1) and SMR analysis depended on the SMR software (v1.3.1)^[24]^. Functional analysis and MAGMA^[20]^ were carried out through the FUMA online annotation platform^[18]^ and corresponding software. Detailed analytical models, assumptions, reference panels, software versions, parameter settings and multiple-testing procedures are provided in **Supplementary Methods S1**.

**Ethical Approval and Consent to Participate**

This study was a secondary cross-trait genetic epidemiology analysis of publicly available GWAS summary statistics. All genetic data utilized in this study were derived from publicly accessible GWAS datasets. No formal ethical approval was obtained by the authors, nor was direct participant consent sought, as all analyses relied on summary-level data from published researches that had already secured appropriate ethical approvals and participant consent.

**Results**

**The Shared Genetic Architecture Between Aging and Constipation-LDSC and HDL**

To begin our investigation into the shared genetic architecture between the FinnGen registry-derived constipation phenotype and systemic aging at a genome-wide level, we estimated their r_g_ using both LDSC and HDL. We found a moderate positive genetic correlation between constipation and FI that remained significant after FDR correction in both LDSC and HDL (LDSC: r_g_ = 0.435, SE = 0.046, 95% CI = 0.345 to 0.525, *P_FDR_* = 1.06 × 10^-20^; HDL: r_g_ = 0.476, SE = 0.051, 95% CI = 0.377 to 0.576, *P_FDR_* = 2.23 × 10^-20^). The inverse association with TL was weaker and method-dependent, remaining significant after FDR correction in LDSC r_g_ = -0.087, SE = 0.037, 95% CI = −0.160 to −0.013, *P_FDR_* = 0.041 but not in HDL r_g_ = -0.101, SE = 0.051, 95% CI = −0.200 to −0.002, *P_FDR_* = 0.092). No significant genome-wide genetic correlation was detected with FA or GAA. The full results of the genetic correlation analyses are detailed in **Table 2 and Supplementary Tables S1-S2**. The cross-trait LDSC intercepts were close to zero(−0.0061 to 0.0017; SEs, 0.0047–0.0055), suggesting limited residual covariance attributable to sample overlap or correlated confounding (**Supplementary Table S1**).

**Identification and validation of pleiotropic risk loci and genes between constipation and aging-related phenotypes**

To systematically characterize potential shared genetic loci between constipation and aging-related phenotypes, we performed PLACO analysis across all prespecified trait pairs. This locus-level analysis was considered complementary to genome-wide genetic correlation analyses, as the two approaches capture different aspects of genetic architecture. We identified 16 pleiotropic SNPs for constipation-FI trait pair, 300 pleiotropic SNPs for constipation-TL and 34 pleiotropic SNPs for constipation-FA, with the complete lists in **Supplementary Tables S3-S5**. FUMA annotation of the genome-wide significant PLACO signals identified 29 distinct genomic risk loci, including 8 for constipation-FA, 1 for constipation-FI, and 20 for constipation-TL. No genome-wide significant pleiotropic locus was retained for constipation-GAA (*P_PLACO_* > 5x10^-8^). At the pairwise Bonferroni sensitivity threshold of *p* < 1.25 × 10⁻⁸, 17 loci remained, including 4 constipation-FA loci and 13 constipation-TL loci (**Supplementary Table S6 and Supplementary Figure S1**).

To further investigate whether the association signals at these loci were consistent with a shared underlying variant rather than distinct signals in linkage disequilibrium, we subsequently performed Bayesian colocalization analysis across the 29 genomic risk loci retained at the prespecified PLACO significance threshold. The full results of this analysis are detailed in **Supplementary Table S7**. We prioritized colocalized loci with strong or suggestive evidence of a shared signal in colocalization analysis for presentation in **Table 3**. Two constipation–FA loci met the stricter criterion for stronger colocalization (Posterior Probability of a shared variant, *PP.H4* > 0.7). Notably, these included the 2p16.1 multi-gene locus mapped to RTN4, CCDC104, SMEK2, PNPT1, and EFEMP1 (*PP.H4* = 0.898) and the 3q13.31 locus containing ZBTB20 (*PP.H4* = 0.706). Furthermore, applying a more lenient exploratory threshold (*PP.H4* > 0.5), we identified suggestive locus-level colocalization at 3q22.3 for the constipation-FI pair (*PP.H4* = 0.543). This multi-gene region includes PPP2R3A, MSL2, PCCB, STAG1, SLC35G2 and NCK1 (**Supplementary Table S8**). **Figure 1** summarizes the relationships among the trait pairs, pleiotropic loci, mapped genes, and colocalization results.

**Evaluation of expression-associated candidate genes using SMR**

To assess gene-expression-associated signals at the candidate loci, we performed SMR analyses using whole-blood eQTL and eQTLGen data. The gene-prioritization evidence was interpreted according to the distinct role of each method (**Figure 2 and Supplementary Table S9**). PLACO identified cross-trait pleiotropic loci, FUMA mapped nearby or functionally annotated genes, MAGMA provided gene-level association evidence, and eQTL data indicated genetic regulation of gene expression. Colocalization assessed whether two trait-association signals were consistent with a shared variant at the locus level, whereas SMR evaluated whether gene-expression and trait associations were compatible with a shared variant-mediated association.

Within each phenotype-by-eQTL dataset analysis, *P_SMR_* were corrected across all tested probes using the Benjamini-Hochberg false discovery rate. Corrected SMR evidence was defined as an FDR-adjusted *P_SMR_* < 0.05 together with *P_HEIDI_* > 0.05, with Bonferroni correction applied as a sensitivity analysis. Although several probes showed nominal associations, none remained significant for constipation in either eQTL dataset after correction. Consequently, no gene showed corrected SMR evidence shared between constipation and any of the four aging-related phenotypes. Signals that survived correction for an aging phenotype alone were interpreted as phenotype-specific expression-associated signals rather than evidence of a shared expression-mediated association. These findings do not exclude locus-level genetic overlap but provide no support for shared mediation through the blood gene-expression signals examined here.

**Gene-based analysis identifies specific pleiotropic genes-MAGMA**

To identify genes showing gene-level association evidence for the cross-trait signals, we used a MAGMA gene-based analysis based on the initial set of shared loci pinpointed by PLACO and FUMA. This approach identified several genes with shared effects on both constipation and aging (**Figure 3; Supplementary Table S10**). The distribution of these findings was as follows: 4 were for the constipation-FI pair, 7 gene associations were linked to the constipation-FA trait pair and 31 were identified for the constipation-TL pair.

**Functional and tissue-specific analyses suggest potential CNS-related involvement**

To characterize the functional context of the identified pleiotropic loci, we performed a MAGMA gene-set enrichment analysis against approximately 17,000 pathways from MSigDB dataset. Across phenotype pairs, three gene sets remained significant after BH-FDR correction. Gene sets related to MECP2-regulated transcription and negative regulation of locomotion were enriched for the constipation–FA pair, whereas a positional gene set at 5p15 was enriched for the constipation–TL pair (**Supplementary Table S11**). Building on this functional evidence, we sought to identify the specific tissues where these genes are predominantly active. A MAGMA gene-property analysis across 54 human tissues highlighted a strong and significant enrichment signal for the genes shared between constipation and FI within the central nervous system, most prominently in the Brain cerebellum (*p* = 2.16x10^-4^, *P_FDR_* = 0.0116, *P_Bonferroni_* = 0.0116) and Brain Cerebellar Hemisphere (*p* = 6.47x10^-4^, *P_FDR_* = 0.0175, *P_Bonferroni_* = 0.0350）, both of which remained significant after BH-FDR and Bonferroni correction. Six tissues were BH-FDR significant for the constipation–TL pair, including ovary, uterus, EBV-transformed lymphocytes, Brain Cerebellar Hemisphere, Brain Cerebellum, and pituitary, although none survived Bonferroni correction. No tissue survived BH-FDR correction for the constipation–FA or constipation–GAA pair (**Figure 4; Supplementary Table S12**). To verify these findings through a different perspective, we used S-LDSC to map heritability across 53 cell types. As MAGMA and S-LDSC capture different dimensions of genetic architecture, we observed partially overlapping but distinct tissue enrichment patterns between the two approaches. S-LDSC revealed significant CNS-related heritability enrichment primarily for FI, including the amygdala, anterior cingulate cortex, and cerebellar hemisphere, whereas constipation showed no significant cell-type enrichment (**Figure 5**). MAGMA identified enrichment in CNS-related tissues for the constipation–FI gene set, whereas S-LDSC detected no significant cell-type enrichment for constipation. These findings highlight candidate CNS-related signals, although their relevance to the shared constipation–FI genetic architecture requires further evaluation.

**Discussion**

Aging is accompanied by systemic and multi-organ decline, which fundamentally shapes the appearance of various diseases^[25][26]^. A prior meta-analysis indicated that the prevalence of Rome-defined functional constipation rises with age, climbing from 12.0% in young adults to 17.0% in those over 60 (OR 1.41;95%CI 1.17-1.70)^[27][28]^. While previous clinical studies have consistently reported a strong association between aging and constipation, the genetic basis of this relationship remains uncertain. We addressed this gap by applying complementary genetic methods to characterize the shared genetic architecture between constipation and aging-related phenotypes. Our analysis identified a consistent positive genetic correlation between constipation and FI and a weaker, method-dependent inverse correlation with TL, whereas no significant genetic correlation was detected with FA or GrimAge acceleration. Through PLACO, FUMA, and Bayesian colocalization, we pinpointed specific pleiotropic loci bridging these traits. Tissue-level analyses suggested that CNS-related tissues may contribute to the shared genetic architecture between constipation and aging-related traits. Among the MAGMA tissue results, the Brain Cerebellum and Brain Cerebellar Hemisphere showed the strongest support for the constipation–FI pair, with both associations surviving BH-FDR and Bonferroni correction (Figure 4). Additional BH-FDR-significant tissue associations were observed for the constipation–TL pair, but none survived Bonferroni correction and they were therefore treated as secondary findings. S-LDSC identified broader CNS enrichment for FI but no significant cell-type enrichment for constipation (Figure 5). These partially distinct findings motivate further functional investigation of CNS-related pathways.

The genetic factors linking constipation to aging are more closely tied to the systemic, multi-system decline captured by FI and TL. Frailty is an aging-linked multidimensional clinical condition, marked by cumulative impairment and gradual decline across multiple systems, greater vulnerability to stress^[29]^. It is associated with a higher likelihood of adverse health outcomes and marked age-associated progression, which elevates the risk of mental disorders, diabetes, cancer and cardiovascular diseases^[30][31][32]^. Clinical studies have reported associations between constipation, frailty, gut-microbiota alterations, nutritional factors, and adverse health outcomes^[33][34]^^[35][36]^. These observations may provide biological context for the constipation–FI genetic correlation. However, the present summary-statistics analyses do not establish an inflammatory, gut–brain, or vitamin B12-mediated mechanism. Our analysis showed a moderate positive genetic correlation between constipation and FI, while a slight negative correlation with telomeres. Telomeres are special protective structures at the end of chromosomes in eukaryotes whose main function is to maintain chromosome integrity and limit the ability of cells to replicate^[37][38]^. Telomere attrition is listed as one of the core hallmarks of aging and can serve as a potential target for pharmacological intervention and treatment^[39][40]^. However, the constipation–TL association observed here was weak and method-dependent and does not provide evidence for a therapeutic target. Recent evidence from Mendelian randomization analysis suggests a potential causal relationship between leukocyte telomere length (LTL) and constipation^[41]^. In zebrafish models, gut-specific telomerase expression (maintaining telomere length) was found to be sufficient to counteract systemic aging, but the precise genetic relationship connecting constipation and TL has not yet been fully elucidated^[42]^. No significant genetic overlap was detected between constipation and FA or GrimAge acceleration. Both FA and constipation are highly heterogeneous traits that are influenced by powerful, independent environmental confounders. Epigenetic clocks function as heritable DNA-based markers that estimate biological age by assessing methylation patterns across a defined set of cytosine–phosphate–guanine (CpG) sites^[43]^. A second representative epigenetic clock, GrimAge integrates DNA methylation with risk factors such as smoking and metabolism^[44][45]^. Its epigenetic signals are more indicative of chronic severe inflammation and metabolic disorder-related pathological processes^[46]^. Even if there is inflammation in constipation, it is usually mild and confined to the intestinal mucosa, and is not sufficient to activate the epigenetic regulatory pathways related to GrimAge acceleration.

To elucidate the genetic connections between constipation and aging-related phenotypes, we employed a multi-stage framework that integrated PLACO, FUMA, colocalization, MAGMA and eQTL annotation. The multi-layer analyses prioritized several candidate genes through different combinations of evidence. The strongest locus-level colocalization evidence was observed for two constipation–FA loci, including a multi-gene locus at 2p16.1 and the ZBTB20-containing locus at 3q13.31. Because the 2p16.1 signal mapped to several genes, it could not be attributed to a single gene. MSL2 and STAG1 were mapped to the same multi-gene constipation–FI locus, which did not meet the stricter cross-pair PLACO threshold and showed only exploratory colocalization (*PP.H4* = 0.543). These genes were therefore retained as candidates requiring further validation rather than robustly supported cross-trait mediators. STAG1 has established roles in chromosome segregation and DNA repair, providing a possible biological context for future investigation of frailty-associated genomic instability^[47][48][49]^. SRSF9 was prioritized for the constipation–TL pair through gene-level and eQTL evidence. Its function as a splicing factor is consistent with the known effects of dysregulation of selective splicing on the aging process provides a possible context for future functional investigation^[50][51]^.

No gene showed multiple-testing-corrected SMR evidence shared between constipation and an aging-related phenotype. This finding does not exclude locus-level pleiotropy or colocalization but provides no support for shared mediation through the blood gene-expression signals examined here. The null result may reflect limited statistical power, tissue-specific gene regulation, or mechanisms not captured by steady-state blood expression. Accordingly, ZBTB20, MSL2, STAG1, SRSF9, and the genes mapped at the 2p16.1 locus should be considered candidates requiring tissue-relevant and functional validation rather than established causal mediators.

MAGMA gene-property analysis indicated expression enrichment in CNS-related tissues among genes prioritized for the constipation–FI pair, with the strongest signals observed in the cerebellum (*p* = 2.16x10^-4^) and cerebellar hemisphere (*p* = 6.47x10^-4^). The cerebellar enrichment observed in MAGMA may provide biological context for further investigation of CNS-related pathways relevant to intestinal function^[52,53]^. Prior studies have also reported age-related changes in gut microbiota^[54]^. However, these observations do not establish a CNS-mediated, brain–gut, or microbiota-mediated mechanism for the constipation–FI association in the present study. Because constipation showed no significant tissue-specific enrichment in S-LDSC, enrichment of the shared genetic component within the CNS remains unconfirmed. Further tissue-resolved genetic and transcriptomic studies may help clarify this relationship.

Several limitations should be considered. First, the constipation phenotype was derived from FinnGen healthcare-register diagnoses and medication-purchase records rather than Rome-based clinical assessment, and the findings may not be specific to functional or chronic idiopathic constipation. Second, all source GWAS datasets were of European ancestry, and the generalizability of our findings to other ancestry groups is therefore uncertain. Differences in allele frequencies and linkage disequilibrium patterns across populations may affect the identification and effect estimates of associated loci and limit the transferability of the observed genetic signals. Future studies using ancestrally diverse GWAS datasets are needed to validate these findings across populations. Exact participant overlap could not be quantified from the available summary statistics, although no known cohort was shared between the FinnGen constipation GWAS and the aging-trait GWAS datasets. Independent replication in an independent European or non-European dataset was not performed in the present study. Third, statistical power varied across phenotype pairs, and the relatively small number of prioritized genes may have limited the stability of the gene-set and tissue analyses. Finally, the analyses were based on summary-level associations, and the differences between MAGMA and S-LDSC tissue findings, together with the negative corrected SMR results, indicate that the candidate genes and CNS-related signals require tissue-relevant functional validation.

**Conclusion**

By integrating genetic correlation, pleiotropic locus mapping, gene-level association, colocalization and tissue-level analyses, we delineated the shared genetic architecture between constipation and selected aging-related phenotypes in European-ancestry populations and prioritized candidate loci, genes and tissues for further investigation. Although shared expression-mediated effects were not supported by the corrected SMR analyses, the convergent locus- and gene-level findings provide a focused framework for functional validation and for investigating the genetic basis of aging-related intestinal dysfunction. These exploratory findings do not establish therapeutic targets, and their potential clinical relevance requires independent replication and functional validation.

# Funding

# The authors received no specific funding for this work

# Competing interests

# The authors declare that they have no conflict of interest.

# Availability of data and material

# All GWAS summary statistics analyzed in this study are publicly available from their original sources, as detailed in Table 1. No individual-level genetic data were accessed or generated in this study. The analysis code is not publicly deposited but is available from the corresponding authors upon reasonable request.

# Author Contributions

# YXL conceived and designed the study, performed data acquisition and analysis, interpreted the data, and drafted the original manuscript. WL contributed to data interpretation. XJL supervised the study. YYX contributed to data analysis and interpretation and substantially revised the manuscript during the revision process. All authors reviewed and approved the final version of the manuscript.

# Ethical Approval and Consent to Participate

This study was a secondary analysis based exclusively on publicly available, anonymized GWAS summary statistics. All original studies that provided data (such as FinnGen and the UK Biobank) had already obtained ethical approval from their respective institutional review boards and written informed consent from all participants. Therefore, no additional ethical approval or consent was required for this analysis. Clinical trial number: not applicable.

**References**

[1]	Mo L, Ma K, Li Y, et al. Dietary fiber from burdock root ameliorates functional constipation in aging rats by regulating intestinal motility[J]. Frontiers in Nutrition, 2025, 12: 1550880. DOI:10.3389/fnut.2025.1550880.

[2]	Scott S M, Simrén M, Farmer A D, et al. Chronic constipation in adults: Contemporary perspectives and clinical challenges. 1: Epidemiology, diagnosis, clinical associations, pathophysiology and investigation[J]. Neurogastroenterology and Motility, 2021, 33(6): e14050. DOI:10.1111/nmo.14050.

[3]	Tang F, Zhao T, Dong P, et al. Association between constipation and risk of coronary heart disease: a systematic review and meta-analysis of cohort studies[J]. *Front Cardiovasc Med.* 2025;12:1622801. doi:10.3389/fcvm.2025.1622801.

[4]	Bharucha A E, Lacy B E. Mechanisms, Evaluation, and Management of Chronic Constipation[J]. Gastroenterology, 2020, 158(5): 1232-1249.e3. DOI:10.1053/j.gastro.2019.12.034.

[5]	Barberio B, Judge C, Savarino E V, et al. Global prevalence of functional constipation according to the Rome criteria: a systematic review and meta-analysis[J]. The Lancet. Gastroenterology & Hepatology, 2021, 6(8): 638-648. DOI:10.1016/S2468-1253(21)00111-4.

[6]	Wang F, Fei M, Hu W Z, et al. Prevalence of Constipation in Elderly and Its Association With Dementia and Mild Cognitive Impairment: A Cross-Sectional Study[J]. Frontiers in Neuroscience, 2021, 15: 821654. DOI:10.3389/fnins.2021.821654.

[7]	De Giorgio R, Ruggeri E, Stanghellini V, et al. Chronic constipation in the elderly: a primer for the gastroenterologist[J]. BMC gastroenterology, 2015, 15: 130. DOI:10.1186/s12876-015-0366-3.

[8]	Rodríguez-Rodero S, Fernández-Morera J L, Menéndez-Torre E, et al. Aging Genetics and Aging[J]. Aging and Disease, 2011, 2(3): 186-195.

[9]	Lai H, Li Y, He Y, et al. Effects of dietary fibers or probiotics on functional constipation symptoms and roles of gut microbiota: a double-blinded randomized placebo trial[J]. Gut Microbes, 2023, 15(1): 2197837. DOI:10.1080/19490976.2023.2197837.

[10]Shashkova T I, Gorev D D, Pakhomov E D, et al. The GWAS-MAP platform for aggregation of results of genome-wide association studies and the GWAS-MAP|homo database of 70 billion genetic associations of human traits[J]. Vavilov Journal of Genetics and Breeding, 2020, 24(8): 876-884. DOI:10.18699/VJ20.686.

[11]Sleigh J W, Morgan P G. Are Genome-wide Association Studies Worth the Trouble?[J]. Anesthesiology, 2024, 141(2): 214-216. DOI:10.1097/ALN.0000000000005074.

[12]Ray D, Chatterjee N. A powerful method for pleiotropic analysis under composite null hypothesis identifies novel shared loci between Type 2 Diabetes and Prostate Cancer[J]. PLoS genetics, 2020, 16(12): e1009218. DOI:10.1371/journal.pgen.1009218.

[13]Mountjoy E, Schmidt E M, Carmona M, et al. An open approach to systematically prioritize causal variants and genes at all published human GWAS trait-associated loci[J]. Nature Genetics, 2021, 53(11): 1527-1533. DOI:10.1038/s41588-021-00945-5.

[14]Subramanian A, Tamayo P, Mootha V K, et al. Gene set enrichment analysis: a knowledge-based approach for interpreting genome-wide expression profiles[J]. Proceedings of the National Academy of Sciences of the United States of America, 2005, 102(43): 15545-15550. DOI:10.1073/pnas.0506580102.

[15]Bulik-Sullivan B, Finucane H K, Anttila V, et al. An atlas of genetic correlations across human diseases and traits[J]. Nature Genetics, 2015, 47(11): 1236-1241. DOI:10.1038/ng.3406.

[16]Ning Z, Pawitan Y, Shen X. High-definition likelihood inference of genetic correlations across human complex traits[J]. Nature Genetics, 2020, 52(8): 859-864. DOI:10.1038/s41588-020-0653-y.

[17]Watanabe K, Taskesen E, van Bochoven A, et al. Functional mapping and annotation of genetic associations with FUMA[J]. Nature Communications, 2017, 8(1): 1826. DOI:10.1038/s41467-017-01261-5.

[18]Giambartolomei C, Vukcevic D, Schadt E E, et al. Bayesian test for colocalisation between pairs of genetic association studies using summary statistics[J]. PLoS genetics, 2014, 10(5): e1004383. DOI:10.1371/journal.pgen.1004383.

[19]Pavlides J M W, Zhu Z, Gratten J, et al. Predicting gene targets from integrative analyses of summary data from GWAS and eQTL studies for 28 human complex traits[J]. Genome Medicine, 2016, 8(1): 84. DOI:10.1186/s13073-016-0338-4.

[20]de Leeuw C A, Mooij J M, Heskes T, et al. MAGMA: generalized gene-set analysis of GWAS data[J]. PLoS computational biology, 2015, 11(4): e1004219. DOI:10.1371/journal.pcbi.1004219.

[21]Finucane H K, Reshef Y A, Anttila V, et al. Heritability enrichment of specifically expressed genes identifies disease-relevant tissues and cell types[J]. Nature Genetics, 2018, 50(4): 621-629. DOI:10.1038/s41588-018-0081-4.

[22]Kurki M I, Karjalainen J, Palta P, et al. FinnGen provides genetic insights from a well-phenotyped isolated population[J]. Nature, 2023, 613(7944): 508-518. DOI:10.1038/s41586-022-05473-8.

[23]FinnGen. K11_CONSTIPATION: Constipation [Internet]. Risteys; [cited 2026 Aug 11]. Available from: https://risteys.finngen.fi/endpoints/K11_CONSTIPATION.

[24]Zhu Z, Zhang F, Hu H, et al. Integration of summary data from GWAS and eQTL studies predicts complex trait gene targets[J]. Nature Genetics, 2016, 48(5): 481-487. DOI:10.1038/ng.3538.

[25]Tian YE, Cropley V, Maier AB, et al. Heterogeneous aging across multiple organ systems and prediction of chronic disease and mortality[J]. Nature Medicine. 2023;29:1221–1231. doi:10.1038/s41591-023-02296-6.

[26]Li Q, Xiao N, Zhang H, et al. Systemic aging and aging-related diseases[J]. FASEB journal: official publication of the Federation of American Societies for Experimental Biology, 2025, 39(5): e70430. DOI:10.1096/fj.202402479RRR.

[27] Kang SJ, Cho YS, Lee TH, et al. Medical Management of Constipation in Elderly Patients: Systematic Review[J]. Journal of Neurogastroenterology and Motility. 2021;27(4):495–512. doi:10.5056/jnm20210.

[28]Suares N C, Ford A C. Prevalence of, and risk factors for, chronic idiopathic constipation in the community: systematic review and meta-analysis[J]. The American Journal of Gastroenterology, 2011, 106(9): 1582-1591; quiz 1581, 1592. DOI:10.1038/ajg.2011.164.

[29]Dent E, Hanlon P, Sim M, et al. Recent developments in frailty identification, management, risk factors and prevention: A narrative review of leading journals in geriatrics and gerontology[J]. Ageing Research Reviews, 2023, 91: 102082. DOI:10.1016/j.arr.2023.102082.

[30]Cao X, Yang Z, Li X, et al. Association of frailty with the incidence risk of cardiovascular disease and type 2 diabetes mellitus in long-term cancer survivors: a prospective cohort study[J]. BMC medicine, 2023, 21(1): 74. DOI:10.1186/s12916-023-02774-1.

[31]Gao L, Di X, Gao L, et al. The Frailty Index and colon cancer: a 2-sample Mendelian-randomization study[J]. Journal of Gastrointestinal Oncology, 2023, 14(2): 798-805. DOI:10.21037/jgo-23-134.

[32]Deng M G, Liu F, Liang Y, et al. Association between frailty and depression: A bidirectional Mendelian randomization study[J]. Science Advances, 2023, 9(38): eadi3902. DOI:10.1126/sciadv.adi3902.

[33]Yan Z, Guan G, Jia H, et al. The association between gut microbiota and accelerated aging and frailty: a Mendelian randomization study[J]. Aging Clinical and Experimental Research, 2025, 37(1): 82. DOI:10.1007/s40520-025-02971-3.

[34]Guo C, Liu S, Liu Y, et al. Development and validation of nomogram for predicting cognitive frailty with multimorbidity: a cross-sectional study[J]. Frontiers in Public Health, 2025, 13: 1606505. DOI:10.3389/fpubh.2025.1606505.

[35]Zhao L, Deng X, Ding N, et al. Diminished representation of vitamin-B12-producing bacteria in constipated elders with frailty[J]. iScience, 2024, 27(8): 110403. DOI:10.1016/j.isci.2024.110403.

[36]Cheever C R, Shams R B, Willingham K R, et al. Understanding constipation as a geriatric syndrome[J]. Geriatric Nursing(New York, N.Y.), 2025, 61: 440-448. DOI:10.1016/j.gerinurse.2024.12.012.

[37]Blackburn E H, Epel E S, Lin J. Human telomere biology: A contributory and interactive factor in aging, disease risks, and protection[J]. Science(New York, N.Y.), 2015, 350(6265): 1193-1198. DOI:10.1126/science.aab3389.

[38]von Zglinicki T. Telomeres: influencing the rate of aging[J]. Annals of the New York Academy of Sciences, 1998, 854: 318-327. DOI:10.1111/j.1749-6632.1998.tb09912.x.

[39]Delrue C, Speeckaert R, Speeckaert M M. Rewinding the Clock: Emerging Pharmacological Strategies for Human Anti-Aging Therapy[J]. International Journal of Molecular Sciences, 2025, 26(19): 9372. DOI:10.3390/ijms26199372.

[40]Rakoczy K, Wojdyło L, Suwała S, et al. Correlations between Aging, Telomeres, and Natural Compounds[J]. Aging and Disease, 2025. DOI:10.14336/AD.2025.0853.

[41]Wang H, Chen X, Wang S, et al. Exploration of the causal effects of leukocyte telomere length and four gastrointestinal diseases: a two-sample bidirectional Mendelian randomization study[J]. BMC gastroenterology, 2023, 23(1): 446. DOI:10.1186/s12876-023-03081-y.

[42]El Maï M, Bird M, Allouche A, et al. Gut-specific telomerase expression counteracts systemic aging in telomerase-deficient zebrafish[J]. Nature Aging, 2023, 3(5): 567-584. DOI:10.1038/s43587-023-00401-5.

[43]Liu Z, Leung D, Thrush K, et al. Underlying features of epigenetic aging clocks in vivo and in vitro[J]. Aging Cell, 2020, 19(10): e13229. DOI:10.1111/acel.13229.

[44]Lu A T, Xue L, Salfati E L, et al. GWAS of epigenetic aging rates in blood reveals a critical role for TERT[J]. Nature Communications, 2018, 9(1): 387. DOI:10.1038/s41467-017-02697-5.

[45]Hillary R F, Stevenson A J, McCartney D L, et al. Epigenetic measures of ageing predict the prevalence and incidence of leading causes of death and disease burden[J]. Clinical Epigenetics, 2020, 12(1): 115. DOI:10.1186/s13148-020-00905-6.

[46]Tian S, Liao X, Chen S, et al. Genetic association of the gut microbiota with epigenetic clocks mediated by inflammatory cytokines: a Mendelian randomization analysis[J]. Frontiers in Immunology, 2024, 15: 1339722. DOI:10.3389/fimmu.2024.1339722.

[47]Fried L P, Tangen C M, Walston J, et al. Frailty in older adults: evidence for a phenotype[J]. The Journals of Gerontology. Series A, Biological Sciences and Medical Sciences, 2001, 56(3): M146-156. DOI:10.1093/gerona/56.3.m146.

[48]Bortz W M. A conceptual framework of frailty: a review[J]. The Journals of Gerontology. Series A, Biological Sciences and Medical Sciences, 2002, 57(5): M283-288. DOI:10.1093/gerona/57.5.m283.

[49]van Wee R, Asor R, Li Y, et al. NIPBL and STAG1 enable loop extrusion by providing differential DNA-cohesin affinity[J]. Proceedings of the National Academy of Sciences of the United States of America, 2025, 122(32): e2514190122. DOI:10.1073/pnas.2514190122.

[50]Bressan G C, Moraes E C, Manfiolli A O, et al. Arginine methylation analysis of the splicing-associated SR protein SFRS9/SRP30C[J]. Cellular & Molecular Biology Letters, 2009, 14(4): 657-669. DOI:10.2478/s11658-009-0024-2.

[51]Fu Y, Huang B, Shi Z, et al. SRSF1 and SRSF9 RNA binding proteins promote Wnt signalling-mediated tumorigenesis by enhancing β-catenin biosynthesis[J]. EMBO molecular medicine, 2013, 5(5): 737-750. DOI:10.1002/emmm.201202218.

[52]Drossman D A, Tack J, Ford A C, et al. Neuromodulators for Functional Gastrointestinal Disorders(Disorders of Gut-Brain Interaction): A Rome Foundation Working Team Report[J]. Gastroenterology, 2018, 154(4): 1140-1171.e1. DOI:10.1053/j.gastro.2017.11.279.

[53]Dinan T G, Cryan J F. The Microbiome-Gut-Brain Axis in Health and Disease[J]. Gastroenterology Clinics of North America, 2017, 46(1): 77-89. DOI:10.1016/j.gtc.2016.09.007.

[54]Liu X, Zhao D, Wu X, et al. Comprehensive 16S rRNA sequencing based microbiomes and 1H NMR based metabolomics reveal the relationships of aging and constipation[J]. Experimental Gerontology, 2022, 166: 111882. DOI:10.1016/j.exger.2022.111882.

**Figures**

**Figure 1: The Shared Genetic Atlas of Constipation and Aging Phenotypes.** This hierarchical plot summarizes the shared genetic architecture, illustrating the connections from constipation (center) to associated aging phenotypes, the pleiotropic genomic loci, and the prioritized candidate genes (outermost layer).

**Figure 2:** **Integrated evidence heatmap for candidate-gene prioritization.** The heatmap visualizes the convergence of evidence for prioritized genes. Rows represent candidate genes identified by the gene-based analysis, with the corresponding aging phenotype shown in parentheses. Columns summarize distinct levels of evidence, including mapping to a pleiotropic locus identified by PLACO and FUMA, eQTL support from eQTLGen or GTEx v8 whole blood, aging-phenotype-specific SMR evidence, and multiple-testing-corrected SMR evidence shared between constipation and the corresponding aging phenotype. Blue tiles indicate that the specified evidence criterion was met, white tiles indicate that the criterion was not met, and grey tiles indicate that the corresponding probe was unavailable in that eQTL resource. SMR P values were corrected across all valid probes separately within each phenotype-by-eQTL-resource analysis using the Benjamini–Hochberg false discovery rate. Corrected SMR support required an FDR-adjusted *P_SMR_* < 0.05 together with a *P_HEIDI_* > 0.05. The age-trait SMR columns indicate evidence for the corresponding aging phenotype alone. Shared SMR evidence required the same gene/probe to meet the corrected SMR and HEIDI criteria for both constipation and the corresponding aging phenotype within the same eQTL resource. No candidate gene met the corrected shared SMR criterion; the same conclusion was obtained using Bonferroni correction as a sensitivity analysis. Locus mapping, eQTL support and phenotype-specific SMR evidence are presented as gene-prioritization evidence and do not establish causal mediation.

**Figure 3: Gene-based Manhattan plot of pleiotropic associations between** **constipation and aging phenotype pairs.** a. Gene-based Manhattan plot of pleiotropic associations between constipation and FI; b. Gene-based Manhattan plot of pleiotropic associations between constipation and FA; c. Gene-based Manhattan plot of pleiotropic associations between constipation and TL. The y-axis represents the gene-level significance. The blue dashed line represents the threshold for suggestive significance (*p* = 1x10^-5^). The red line indicates the pair-specific Bonferroni significance threshold: *p* = 2.7522 × 10^-6^ for constipation–FI (18,167 genes), *p* = 2.7021 × 10^-6^ for constipation–FA (18,504 genes) and *p* =2.6817 × 10^-6^ for constipation–TL (18,645 genes). Genes exceeding the corresponding Bonferroni threshold are labelled with their official symbols.

**Figure 4: Tissue-specific enrichment of genes shared between constipation and aging-related phenotypes.** The bar plot displays the results of a MAGMA gene-property analysis testing for the enrichment of shared genes across 54 human tissues from the GTEx project. The blue dashed line denotes the nominal significance threshold (*p =* 0.05), whereas the red dashed line denotes the Bonferroni threshold of *p* < 9.26 × 10^-4^ (0.05/54 GTEx tissues). Asterisks indicate tissues that remained significant after BH-FDR correction within the corresponding phenotype pair.

**Figure 5: S-LDSC tissue-specific heritability enrichment across constipation and aging-related phenotypes.** Columns represent constipation, FI, FA, TL, and GAA, and rows represent 53 GTEx tissues. Darker blue indicates a smaller *p* value, and asterisks denote BH-FDR < 0.05 within each phenotype. S-LDSC assesses phenotype-specific rather than cross-trait enrichment.

**Supplementary file legends**

**Supplementary Methods S1.** Core analytical models and parameter settings.

**Supplementary Table S1.** Genetic correlation analysis conducted by LDSC.

**Supplementary Table S2.** Genetic correlation analysis conducted by HDL.

**Supplementary Table S3.** Genome-wide significant pleiotropic variants identified by PLACO for constipation and facial aging.

**Supplementary Table S4.** Genome-wide significant pleiotropic variants identified by PLACO for constipation and telomere length.

**Supplementary Table S5.** Genome-wide significant pleiotropic variants identified by PLACO for constipation and frailty index.

**Supplementary Table S6.** Genomic risk loci between constipation and aging-related phenotypes.

**Supplementary Table S7.** Bayesian colocalization results for the 29 pleiotropic genomic risk loci retained under the primary PLACO threshold.

**Supplementary Table S8.** FUMA-annotated pleiotropic loci with Bayesian colocalization results.

**Supplementary Table S9.** Summary of multi-layered evidence for candidate genes linking constipation and aging.

**Supplementary Table S10.** Bonferroni-significant MAGMA gene-based results for each constipation–aging phenotype pair.

**Supplementary Table S11.** Top-ranked MAGMA gene-set results for each constipation–aging phenotype pair.

**Supplementary Table S12.** Complete MAGMA gene-property analysis across 54 GTEx tissues for each constipation–aging phenotype pair.

**Supplementary Figure S1.** PLACO sensitivity analysis. Numbers of significant variants and genomic risk loci at the primary *P_PLACO_* < 5 × 10^-8^ and cross-pair Bonferroni *P_PLACO_* < 1.25 × 10^-8^ thresholds. The stricter threshold retained 17 of 29 loci, including 4 constipation–FA and 13 constipation–TL loci.
